# Effect of Potato-Derived Ingredients on the Quality Characteristics and Resistant Starch Content of Durum Wheat Pasta

**DOI:** 10.3390/foods15203701

**Published:** 2026-10-17

**Authors:** Burcu Aykanat, Paulina Łysakowska, Piotr Zarzycki, Aldona Sobota

**Affiliations:** 1 Department of Agricultural, Forestry, Food and Environmental Sciences (DAFE), University of Basilicata, Via Dell’Ateneo Lucano 10, 85100 Potenza, Italy; 2 Department of Engineering and Cereal Technology, University of Life Sciences in Lublin, Skromna 8, 20-704 Lublin, Polandpiotr.zarzycki@up.edu.pl (P.Z.)

**Keywords:** functional pasta, starch digestibility, dietary fiber, texture profile analysis, cooking quality, cereal products, starch modification

## Abstract

The incorporation of potato-derived ingredients into cereal-based products represents a promising strategy for improving their nutritional quality while promoting resistant starch (RS) formation through starch retrogradation. This study investigated the effects of potato starch (PS), potato flakes (PF), and potato grits (PG), incorporated at 10 and 20% substitution levels, on the technological, physicochemical, nutritional, and RS characteristics of durum wheat pasta. All potato-enriched pasta samples were compared with a control pasta (CON) produced from 100% durum wheat semolina without any additives. Pasta samples were evaluated for farinograph properties, cooking quality, texture profile, color, chemical composition, and starch digestibility after cooking, cooling (4 °C, 24 h) and freezing (−18 °C, 24 h) treatments. The incorporation of potato materials significantly affected dough behavior and pasta quality depending on the type and level of supplementation. The incorporation of PF and 20% PG slightly increased total dietary fiber (TDF) content in pasta samples, although the differences were not statistically significant (*p* > 0.05), whereas PS addition reduced TDF levels.Potato-enriched pasta exhibited shorter minimal cooking times (MCT) than the control, while cooking loss (CL) increased, particularly in PG20 (9.33% d.b.). Texture properties were strongly influenced by the potato ingredient, with PF producing the highest hardness and adhesiveness values, whereas PS showed textural characteristics closer to those of the control. Cooling alone resulted in negligible RS formation in most samples; however, frozen storage resulted in substantially higher RS contents than refrigerated storage, a response consistent with the formation of retrograded, enzyme-resistant starch structures. The highest RS content was observed in PG20 (3.18% d.b.), representing a 335.6% increase compared with the control, accompanied by a reduction in digestible starch. These findings demonstrate that the physical form of potato ingredients plays a key role in determining pasta quality and that PG, combined with frozen storage, may increase the RS content of durum wheat pasta, although this was accompanied by higher cooking loss and changes in texture.

## 1. Introduction

Durum wheat pasta is a widely consumed staple food valued for its affordability, long shelf life, ease of preparation, and versatility [1]. As an established component of the Mediterranean diet, pasta generally produces a lower glycemic response than many other refined cereal products. This response is largely attributed to its dense gluten–starch matrix, which limits enzyme accessibility and slows starch hydrolysis during digestion [1,2]. Nevertheless, conventional pasta consists mainly of digestible starch and contains relatively little dietary fiber. Growing demand for foods with improved nutritional and physiological properties has therefore encouraged the enrichment of pasta with functional ingredients. However, replacing semolina with potato-derived ingredients can alter water distribution, gluten development, starch gelatinization, cooking behavior, and texture. Pasta reformulation must therefore balance nutritional improvement with the preservation of technological quality [2,3].

Resistant starch (RS), defined as the fraction of starch that resists digestion in the small intestine and arrives in the colon, is particularly relevant to this approach [4]. RS is classified into five types (RSI–RSV) according to its structure and resistance to enzymatic digestion, including physically inaccessible starch, native resistant granules, retrograded starch, chemically modified starch, and amylose–lipid complexes. Interactions between amylose and other food components may also limit starch digestibility [5,6]. Among these fractions, RSIII is especially important in cooked foods because it can be generated during cooling and storage. Its ordered structure limits α-amylase activity, thereby reducing the amount and rate of glucose released during digestion [7]. RS that reaches the colon can also be fermented by the intestinal microbiota, producing short-chain fatty acids such as acetate, propionate, and butyrate. These metabolites contribute to colonic health and may support intestinal barrier function, microbial balance, glucose homeostasis, and lipid metabolism. However, the physiological effects of RS depend on its structure, dose, food matrix, and processing history [5,6,8].

Potato-derived ingredients are promising sources of starch and other nutrients for cereal-based foods. Potato starch has relatively large granules, high swelling capacity, and strong paste-forming properties, partly because of the phosphate groups naturally present in amylopectin [9,10,11]. Unlike isolated starch, potato flour retains other tuber constituents, including protein, fiber, and mineral matter [12]. Potato flour, flakes, and grits may behave differently in a food matrix because their production involves different combinations of thermal pretreatment and mechanical operations. These processing conditions can alter particle morphology and porosity, starch granule integrity, cellular structure, and the extent of starch gelatinization, thereby affecting the functional properties of the resulting potato ingredients [13,14,15]. For example, potato flakes contain pregelatinized starch and rapidly develop viscosity during hydration, whereas less extensively processed materials such as potato flour may retain more intact starch granules [11,16]. Drying and pretreatment also influence the chemical, structural, and pasting properties of potato flour and, consequently, its behaviour during dough development and cooking [12,15,17]. The functionality of a potato ingredient is therefore determined not only by its composition but also by its processing history and physical form.

Previous studies have shown that incorporating potato ingredients can substantially alter wheat-based doughs and products. Potato starch changes water distribution, interferes with gluten aggregation, and affects the rheological and cooking properties of fresh noodles [10]. Model-dough experiments have similarly shown that potato starch interacts less effectively with gluten than wheat starch, resulting in a less stable viscoelastic structure [18]. In potato flour–wheat flour systems, gluten dilution and competition for water progressively reduce network continuity as the substitution level increases [19,20]. This effect can be particularly pronounced with highly gelatinized ingredients. The strong water absorption of potato flakes can restrict gluten hydration and expose gliadin-rich regions associated with increased dough stickiness [16]. Potato-flour processing method and particle size have also been shown to influence dough development, pasting behaviour, color, cooking loss, texture, and sensory acceptance in noodles [15,17].

Similar effects have been reported in pasta. Sharma et al. [21] observed changes in cooking quality and in vitro starch digestibility following the incorporation of potato and pigeonpea flours, although the individual contribution of potato flour could not be fully distinguished from that of the legume component. Sharma et al. [22] subsequently demonstrated that potato flour and potato mash produced different effects on pasting properties, cooking loss, texture, microstructure, and starch digestibility, highlighting the importance of ingredient form. The incorporation of sweet potato starch, banana starch, debranched waxy rice starch, and potato-peel fractions has likewise shown that increasing the RS or fiber content of pasta is often accompanied by changes in cooking time, water uptake, firmness, stickiness, and dry matter loss during cooking [23,24,25,26]. These findings demonstrate that the nutritional effects of pasta enrichment must be considered alongside the structural changes caused by semolina replacement.

Post-cooking storage promotes RSIII formation through the reassociation and recrystallization of gelatinized starch chains during cooling. Amylose retrogrades more rapidly than amylopectin, while the extent of retrogradation depends on starch composition, moisture availability, interactions with other food components, and previous processing conditions [5,27,28].

Refrigeration can provide sufficient molecular mobility for starch-chain reassociation, although its effect depends on the food matrix. Cold storage has reduced starch digestibility in some pasta studies, while producing only modest increases in RS in others [29,30]. For instance, Kolarič et al. [24] reported an increase in RS after sweet-potato-starch pasta was stored for 24 h. Storage temperature, duration, and residual moisture have also been shown to affect molecular organization, enzyme resistance, and estimated glycemic response in cooked noodles [31]. Freezing produces a more complex response. Ice formation removes water from the unfrozen phase, locally concentrates starch polymers, and may disrupt the cooked matrix. At the same time, low temperatures restrict molecular mobility. Consequently, storage at −18 °C does not necessarily promote greater RSIII formation than refrigeration, as the outcome depends on available water, freezing rate, ice-crystal formation, starch-chain mobility, and structural changes during thawing. Yi et al. [32] found that storage at 4 °C reduced starch digestibility in cooked rice more effectively than storage at −18 °C, although the response varied with starch molecular structure.

Despite increasing interest in potato-enriched cereal products, direct comparisons of potato starch, flakes, and grits under identical pasta-processing conditions remain limited. Previous studies have generally investigated individual potato ingredients, substitution levels, or botanical starch sources. Less attention has been given to the combined influence of ingredient form and post-cooking storage temperature. It therefore remains unclear whether potato starch, flakes, and grits produce comparable changes in the gluten–starch matrix or respond similarly to refrigeration and freezing. In particular, their respective effects on water distribution, starch gelatinization, matrix continuity, retrogradation, and the balance between RS formation and technological quality have not been established.

This study aimed to evaluate the effects of replacing durum wheat semolina with three potato-derived ingredients at 10% and 20% on pasta quality and physicochemical properties. The farinograph properties of the blends, pasta composition, cooking quality, color, texture, and RS content after storage at 4 °C or −18 °C for 24 h were assessed. The novelty lies in comparing potato ingredients differing in processing intensity and physical characteristics within the same pasta system and evaluating their effects on pasta quality and RS formation during post-cooking storage.

## 2. Materials and Methods

### 2.1. Raw Materials

Durum wheat semolina (DWS) was supplied by Mulino Caputo (Antimo Caputo S.R.L., Napoli, Italy). Three commercially available potato materials were used: potato starch (PS) supplied by PPH “JANEX” J. Ciupak, L. Ciupak Sp. J. (Janów Lubelski, Poland), and potato flakes (PF), and potato grits (PG) supplied by Zakłady Przemysłu Ziemniaczanego Sp. z o.o. (Lublin, Poland). The potato-derived ingredients differed in their physical characteristics and degree of processing. Potato starch (PS) consisted predominantly of starch granules, whereas potato grits (PG) were obtained by peeling, blanching, drying, and grinding potato material. Potato flakes are obtained by cooking and mashing potatoes, followed by drum-drying the puree and breaking the dried products into flakes.

The raw materials were characterized in terms of particle size distribution [1], degree of starch gelatinization (PN-A-79011-11:1998), water absorption index (WAI) and water solubility index (WSI) [33], as well as total starch and resistant starch (RS) contents [33]. Particle size distribution was determined by sieve analysis using sieves with mesh sizes of 400, 315, 250, 200, 160, 125, and 80 µm for potato grits, potato flakes, and durum wheat semolina. For potato starch, an additional sieve with a mesh size of 60 µm was used.

### 2.2. Determination of Pasting Characteristics of Raw Materials

To determine the pasting properties, the pasting characteristics of aqueous suspensions of potato-derived raw materials (PS, PG, and PF) and durum wheat semolina were determined using a Rapid Visco Analyzer (RVA) approach. Aqueous suspensions of the above-mentioned raw materials were prepared at a concentration of 10%. The measurements were performed using an oscillatory-rotational rheometer, MCR 92 (Anton Paar, Graz, Austria). Before the measurements, the prepared suspensions were mixed at 960 rpm for 10 s. Subsequently, the stirring speed was reduced to 160 rpm. The samples were equilibrated at 50 °C for 1 min and then heated from 50 to 95 °C at a rate of 2.5 °C/min. The samples were held at 95 °C for 15 min and subsequently cooled to 50 °C at a rate of 2.5 °C/min and held at 50 °C for 2 min.

Based on the obtained curves, the following parameters were determined: peak viscosity, peak time, pasting temperature, holding strength, holding time, final viscosity, breakdown (the difference between peak viscosity and holding strength), setback region (the difference between final viscosity and peak viscosity), and setback (the difference between final viscosity and holding strength). The measurements were performed in triplicate for each flour and each suspension concentration.

### 2.3. Farinograph Characteristics of Semolina–Potato Material Blends

Farinograph measurements were conducted on semolina and semolina–potato material blends using a Farinograph-E (model 8110.142, Brabender, Duisburg, Germany). The tests followed ICC Standard No. 115/1 [34], AACC Method 54-21.02. Dough development time (DDT, min), water absorption (%), dough stability (min), degree of softening after 12 min (FU), and farinograph quality number (FQN, mm) were determined.

### 2.4. Pasta Production

Pasta samples were produced on a semi-technical laboratory scale using a MAC 30S-Lab extruder (ItalPast, Fidenza, Italy). Potato raw material substitution levels were selected based on preliminary pilot-scale trials. In this study, a control sample containing only DWS was partially substituted with each raw potato material on a weight basis at 10% and 20%. For each formulation, a 3 kg batch of dry ingredients was mixed with the amount of water required to achieve a final dough moisture content of 32%.

After 15 min of premixing, the dough was vacuum-extruded through a Teflon die to produce Fusilli-shaped pasta. Barrel temperature and extrusion pressure were recorded during processing. The pasta samples were then dried for 13 h in an EAC 30-Lab chamber dryer (ItalPast, Fidenza, Italy) using a low-temperature cycle during which the temperature was gradually reduced from 55 to 35 °C and relative humidity (RH) from 75% to 55%. The dried pasta samples were packed in plastic bags and stored at 4 °C until analysis. Pasta formulations and processing parameters, including extrusion pressure and extruder capacity, are summarized in Table 1.

### 2.5. Determination of Starch Digestibility

Resistant starch (RS) content was determined using an enzymatic method with the Resistant Starch Assay Kit (K-RSTAR, Megazyme, Neogen Corporation, Lansing, MI, USA), according to the recommended protocol (version 08/23, AOAC 2002.02; AACC 32-40.01). For each pasta sample, three 50 g portions were prepared and cooked for the predetermined minimum cooking time (MCT). After cooking, the samples were drained and cooled for 15 min at 25 °C. One portion was subsequently dried at 55 °C for 24 h, while the second and third portions were stored at 4 °C and −18 °C, respectively. After 24 h of refrigerated or frozen storage, the samples were dried at 55 °C for 24 h. The dried samples were ground using a Laboratory Mill 120 (Perten Instruments AB, Stockholm, Sweden), packed in plastic bags, and stored under refrigerated conditions until analysis. Moisture content was determined for the prepared samples, and the RS content was expressed on a dry-matter basis. The cooking procedure for each pasta sample was performed in duplicate.

### 2.6. Chemical Analysis

The chemical composition of raw materials and dried pasta samples was determined by measuring moisture (AACC Method 44-15A), ash (AACC Method 08-01), crude protein (AACC Method 46-08), crude fat (AACC Method 30-10), and total dietary fiber (TDF; AACC Methods 32-05 and 32-21; AOAC Methods 985.29 and 991.43). Digestible carbohydrates were calculated by difference from the combined mass of protein, fat, TDF, and ash in 100 g of dry matter of pasta or raw material.

Protein content was measured by the Kjeldahl method using a Kjeltec™ 9 Analyzer (FOSS, Copenhagen, Denmark). Total nitrogen content was converted to protein using a factor of 5.7 for DWS and pasta samples and 6.25 for the potato materials. Fat content was determined by continuous solvent extraction using a Soxtec™ 8000 system (FOSS, Copenhagen, Denmark).

Total dietary fiber (TDF), insoluble dietary fiber (IDF), and soluble dietary fiber (SDF) were determined by enzymatic-gravimetric methods using Megazyme (Bray, Co. Wicklow, Ireland) enzymatic assay kits. TDF was calculated as the sum of the IDF and SDF fractions.

The energy value of each pasta sample was calculated using the modified Atwater coefficients (protein—4 kcal/g, carbohydrates—4 kcal/g, fat—9 kcal/g, and TDF—2 kcal/g).

### 2.7. Cooking Properties of Pasta Samples

Cooking quality of pasta samples was evaluated by measuring minimum cooking time (MCT, min), weight increase index (WII), and cooking loss (CL) using the standard methods of AACC Method 66-50.01. Exactly 50 g of pasta samples was cooked in 600 mL of boiling distilled water (95–100 °C).

MCT was defined as the time required for the white core to disappear completely when a pasta piece was compressed between two glass slides. The WII was determined as a ratio between the mass of cooked and uncooked pasta. CL was assessed by determining the dry matter content in water after cooking for the MCT.

### 2.8. Texture Profile Analysis

The texture of cooked pasta was assessed using a Texture Profile Analysis (TPA) test performed with a Brookfield AMETEK CTX texture analyzer (Middleboro, MA, USA), equipped with a cylindrical probe (TA4/1000) with a diameter of 38.1 mm. Pasta samples were cooked to their OCT, immediately cooled under running tap water to terminate the cooking process, and equilibrated to room temperature before the measurements. For each measurement, an individual cooked pasta sample was placed centrally on the instrument platform and subjected to two consecutive compression cycles. The test was performed with a trigger force of 0.05 N, a deformation level of 50%, and a probe speed of 1 mm·s^−1^. Hardness (N), adhesiveness (N·s), cohesiveness (N), and springiness index were determined from the force–time curves. The measurement was repeated five times for each sample (*n* = 5), and the results were expressed as mean values ± standard deviation.

### 2.9. Color Parameters of Pasta Samples

The whole fusilli pasta was coarsely ground in a mortar to obtain uniform, fine-grained fragments. The crushed sample was then transferred into the colorimeter’s measuring cup, thoroughly packed by shaking it several times to ensure complete filling. Color parameters of dry crushed pasta were evaluated in the CIE Lab* system using a Chroma Meter CR-5 spherical spectrophotometer (Konica Minolta, Sakai, Osaka, Japan). The instrument was calibrated with white and black reference plates before analysis. Measurements were performed under a D65 illuminant using a 10° standard observer and an 8 mm aperture. Ten measurements were recorded for each formulation (*n* = 10).

The total color difference (ΔE*) between the control and potato-enriched pasta samples was calculated according to Kolarič et al. [24].

$$\Delta E^{*}=\sqrt{{\left(\Delta L^{*}\right)}^{2}+{\left(\Delta a^{*}\right)}^{2}+{\left(\Delta b^{*}\right)}^{2}}$$

where ΔL^*^, Δa^*^, *and* Δb^*^ represent the differences between the respective color coordinates of the control and potato-enriched pasta samples.

Whiteness index (*WI*) and yellowness index (*YI*) were also calculated.

$$\mathrm{WI}=100-\sqrt{{\left(100-L^{*}\right)}^{2}+{\left(a^{*}\right)}^{2}+{{(b}^{*})}^{2}}$$

$$\mathrm{YI}=142.86\times \frac{b^{*}}{L^{*}}$$

### 2.10. Statistical Analysis 

Results were reported as mean values ± standard deviation of analytical replicates. Differences among pasta formulations were evaluated by one-way analysis of variance (ANOVA), followed by Tukey’s post hoc test, for multiple comparisons. Comparisons between two groups were performed using an independent-samples *t*-test. Differences were considered statistically significant at *p* < 0.05. Since each formulation was produced as a single batch, the replicates represent analytical rather than independent experimental replicates. Farinograph measurements and chemical composition analyses were performed in duplicate (*n* = 2); their coefficients of variation were below 1% for farinograph water absorption and below 3% for moisture and protein content, whereas higher variability was observed for the degree of softening and the dietary fiber fractions, so differences in these parameters are interpreted with caution. Statistical analyses were performed using OriginPro 2025b software (OriginLab Corporation, Northampton, MA, USA). 

## 3. Results and Discussion

### 3.1. Physicochemical Characteristics and Functional Properties of Raw Materials

Table 2 presents the physicochemical and functional properties of the raw materials (PS, PG, PF, and DWS). Potato starch was characterized by the smallest mean particle size, with 100% of the particles belonging to the fine fraction (<160 µm). The largest equivalent particle diameters were observed for PG (336.89 µm) and PF (302.61 µm), whereas the corresponding value for DWS was 155.77 µm.

PF exhibited the highest degree of starch gelatinization (98.04%), confirming the high degree of thermal processing of this raw material. A lower degree of this parameter was observed for PG (76.4%) and DWS, while the lowest value was recorded for PS.

At the same time, PF were characterized by the highest WAI (712.14%) and WSI (30.79%). For comparison, the WSI of PS was almost 27-fold lower, while its WAI was 4.03-fold lower.

PS was characterized by the highest total starch content (85.54% d.b.), including resistant starch (RS = 22.48% d.b.). In contrast, the lowest levels of resistant starch were recorded in potato flakes (2.05% d.b.) and durum wheat semolina (2.39% d.b.).

Analysis of the pasting properties of the tested potato raw materials and DWS demonstrated significant variation in pasting kinetics, viscosity, and thermomechanical stability of the resulting pastes (Table 3).

Pasting temperature marks the onset of starch granule swelling and ranges within a narrow interval from 54.4 °C for PF to 56.3 °C for DWS. The lowest values were recorded for PF (54.4 °C) and native PS (55.53 °C). The lower pasting initiation temperature of potato raw materials is associated with weaker intramolecular interactions within potato starch granules and the presence of phosphate ester groups, which increase molecular hydrophilicity [35].

A key indicator of the degree of prior hydrothermal modification is the time to reach maximum viscosity (Peak Time). PF is characterized by a drastically reduced Peak Time of only 1.03 min. Such a short duration confirms the presence of pre-gelatinized starch resulting from thermal processing during flake production. Pre-gelatinized starch enables immediate water binding without requiring prolonged heating. For comparison, PS reaches its peak only after 12.43 min, whereas PG and DWS require 25.7 min and 24.2 min, respectively, for full structural development.

Peak Viscosity reflects the free swelling capacity of starch granules prior to mechanical disintegration. Potato starch (PS) exhibited the highest peak viscosity value (8350.33 cP), exceeding the results for PF (2865.0 cP), PG (1426.1 cP), and DWS (888.7 cP). The extremely high peak viscosity of PS serves as direct evidence of a low degree of initial gelatinization in this raw material, as confirmed by the research results presented in Table 2. Potato starch granules are characterized by larger size and looser crystalline packing compared to wheat starch in DWS, whose swelling is further constrained by the gluten matrix.

The Breakdown index, defined as the difference between peak viscosity and viscosity at the holding phase (Holding Strength), measures the susceptibility of gelatinized granules to destruction under shear stress and elevated temperature. PS achieved a very high Breakdown value (6656.67 cP). Significantly lower breakdown values (*p* < 0.05) were recorded for PF (2073.0 cP), PG (182.6 cP), and DWS (169.5 cP), respectively. Such a high viscosity drop in PS and PF samples confirms that potato starch granules, upon reaching maximum swelling, readily undergo disintegration and structural disruption under shearing. Conversely, the low Breakdown values for PG and DWS indicate higher structural integrity of these raw materials (due to the presence of cell walls, and in semolina, additionally the protein matrix).

The cooling process of the paste leads to amylose recrystallization and the formation of a continuous gel network, which is described by Final Viscosity and Setback parameters. PS achieved the highest final viscosity (2706.33 cP) and the highest Setback index (1012.3 cP). For DWS, the setback index was 661.7 cP, indicating a pronounced tendency of the amylose present in this raw material to re-associate. PF and PG samples obtained lower Setback values (429.3 cP and 326.9 cP, respectively).

### 3.2. Pasta Processing

The type of potato-derived ingredient had a clear effect on pasta processing behavior, whereas increasing the substitution level did not produce a uniform response across the three materials (Table 1). All formulations were processed at the same vacuum pressure in the mixing chamber (0.08 MPa), while barrel temperature remained within a narrow range of 28.5–29.7 °C. The most pronounced effect was observed for PS, which significantly increased (*p* < 0.05) extruder capacity from 24.60 kg h^−1^ in the control to 28.26 and 30.48 kg h^−1^ at 10 and 20% substitution, respectively. In contrast, the capacities obtained with PF and PG (25.14–26.16 kg h^−1^) did not differ significantly from the control (*p* > 0.05). Extrusion pressure also varied according to ingredient type, decreasing from 18 MPa in the control to 12–14 MPa for PF and 14–16.5 MPa for PG, whereas PS showed values of 15.5 and 18 MPa at 10 and 20% substitution, respectively.

The higher processing capacity observed with PS may be related to the modification of dough resistance caused by partial replacement of the gluten-forming semolina fraction with a predominantly starch-based ingredient. Incorporation of potato starch into wheat dough has been shown to reduce the continuity of the gluten network and modify its viscoelastic behavior, because the added starch both dilutes gluten proteins and alters competition for water within the matrix [10]. Microscopic observations reported by Tao et al. [10] showed progressively greater discontinuities in the gluten network as potato starch substitution increased, while the elastic and viscous characteristics of the dough were also modified. Such structural weakening may reduce resistance to material transport through the extruder, which could partly explain the increased throughput of the PS formulations.

The different response of PF and PG suggests, however, that gluten dilution alone cannot account for the processing behavior of the enriched doughs. Unlike the highly starch-rich PS material, flakes and grits contain additional potato matrix components and showed greater water requirements during farinograph analysis, as discussed in Section 3.3. Under the fixed dough moisture used for pasta manufacture, this stronger hydration demand may have reduced the amount of freely available water and partially counteracted the reduction in structural resistance expected from gluten dilution. Similar matrix-dependent effects have been observed in potato-enriched cereal systems. Sharma et al. [21] attributed changes in potato-containing pasta to the combined influence of starch structure, water affinity and modifications of the three-dimensional protein–starch matrix rather than to the level of substitution alone. Likewise, incorporation of sweet-potato starch into durum wheat dough altered rheological behavior through simultaneous changes in gluten content and starch hydration, with the magnitude of the response depending on the physicochemical characteristics of the added starch [24]. The contrasting extrusion behavior of PS, PF and PG therefore indicates that the nature and previous processing of the potato ingredient, rather than simply its proportion in the formulation, governed its interaction with the semolina matrix during pasta manufacture.

### 3.3. Farinograph Characteristics of Semolina-Potato Blends

The most pronounced effect of potato supplementation was the contrasting hydration behavior of the three ingredients (Table 4). Water absorption of the control semolina was 61.45%, and substitution with PS progressively reduced this value to 59.35 and 57.30% at 10 and 20%, respectively. In contrast, PF and PG significantly (*p* < 0.05) increased water absorption, reaching 76.30 and 85.95% for PF10 and PF20 and 71.60 and 81.30% for PG10 and PG20. All formulations differed significantly (*p* < 0.05) for this parameter, showing that dough hydration was governed primarily by the nature of the potato ingredient rather than by substitution level alone.

The exceptionally high water requirement of the PF blends is consistent with the physicochemical behavior of processed potato flakes. Wang et al. [16] reported that potato flakes exhibited substantially greater water-absorption capacity than both gluten and wheat starch and demonstrated that this strong hydration competed with gluten proteins for available water, resulting in a more discontinuous gluten network. This behavior is associated with the processing history of flakes, during which starch undergoes partial or extensive gelatinization, increasing accessibility of hydrophilic groups and promoting rapid hydration. Similar effects were observed by Buzera et al. [17], who found that the water absorption of wheat–potato blends depended strongly on the potato-flour preparation method; flour subjected to blanching and subsequent drying showed greater hydration than freeze-dried material, which the authors attributed partly to differences in starch gelatinization. The particularly high absorption of PF in the present study is therefore consistent with a more highly processed potato matrix containing starch with enhanced hydration capacity.

PG also substantially increased water absorption, although values remained below those of the corresponding PF formulations. This difference may reflect a lower extent of starch disruption or gelatinization and greater preservation of cellular structure in the grits, which would restrict the immediate accessibility of starch to water compared with flakes. The importance of processing history is supported by Buzera et al. [17], who showed that two potato flours produced from the same raw material but using different pretreatments differed considerably in particle size, farinograph behavior and subsequent noodle quality. Thus, the differences between PF and PG are more plausibly related to their structural state than simply to their common botanical origin.

In contrast to PF and PG, incorporation of the starch-rich PS fraction, containing predominantly ungelatinized starch, reduced farinograph water absorption. A similar decrease has been reported by Kolarič et al. [24] for durum wheat dough enriched with sweet-potato starch, where increasing starch substitution progressively reduced the water required to attain the target dough consistency. This behavior can result from dilution of the gluten-forming protein fraction and the resulting change in the relative contributions of protein and starch to water binding. However, literature findings for isolated potato starch are not completely uniform. Xu et al. [18], using controlled potato starch–gluten model systems, found higher water retention and Mixolab water absorption for potato starch than for wheat starch and also demonstrated that potato starch interacted less favorably with gluten than wheat starch. The apparent discrepancy indicates that farinograph water absorption in a complete cereal dough cannot be predicted from starch water-holding capacity alone; gluten concentration, starch damage, granule size, starch source and the distribution of water between matrix components all contribute to the measured response. Recent work on wheat dough has similarly shown that hydration state is a major determinant of gluten-network morphology and rheological behavior [36].

The different hydration patterns were accompanied by changes in dough development. PS had little effect, with development times of 1.87 and 1.85 min compared with 1.83 min for the control, while PF produced only a moderate numerical increase to 2.27–2.52 min. In contrast, PG significantly (*p* < 0.05) prolonged development time of dough to 6.43 and 7.21 min at 10 and 20% substitution, respectively. The longer development time of PG should not be interpreted as stronger gluten formation because its dough stability decreased concurrently. Rather, the prolonged mixing requirement is more consistent with slower hydration and redistribution of water in a heterogeneous semolina–grit system. This may be related to the presence of fragments of cellular structures in the potato grits, which can hinder water penetration and prolong the hydration of the raw materials, thereby potentially delaying gluten network development. Processing-dependent increases in development time have also been reported by Buzera et al. [17], who observed substantially longer dough development when wheat flour was supplemented with blanched–oven-dried potato flour than with freeze-dried potato flour, with development time increasing further at higher incorporation levels. This provides direct evidence that the physical state of potato material can determine how rapidly the composite dough reaches maximum consistency.

The weakening effect of potato supplementation was most evident from dough stability and softening. The control showed a stability time of 15.37 min, whereas PF10 decreased this value to only 1.25 min. PG caused a less severe but concentration-dependent reduction, from 8.44 min at 10% to 5.13 min at 20%. In comparison, PS10 retained a stability time of 14.54 min, close to that of the control; the corresponding value for PS20 was not reported. The degree of softening followed the same overall pattern among the available measurements, increasing from 25 FU in the control to 212 FU for PF10 and to 88.5 and 128.5 FU for PG10 and PG20, whereas PS remained close to the control.

The severe reduction in stability of PF is consistent with competition for water between the potato matrix and gluten proteins. Wang et al. [16] directly observed that the high hydration capacity of potato flakes restricted gluten hydration and generated a discontinuous protein network. Hu et al. [20] reached a complementary conclusion for potato–wheat dough, showing that water binding by potato-derived dietary fiber partially dehydrated gluten proteins, while insoluble cellulose imposed steric restrictions on development of the continuous gluten phase. These mechanisms are particularly relevant to PF and PG, which retain considerably more non-starch potato material than the starch-rich PS ingredient.

Recent studies with potato flour and potato powder also report reduced dough stability and progressive deterioration of gluten organization as the replacement level increases. Yang et al. [19] observed decreases in stability, development time and viscoelastic moduli in wheat–potato powder doughs and attributed these effects to gluten dilution together with competition for water between starch, fiber and protein. In model systems, Xu et al. [18] similarly found that potato starch exerted a stronger disruptive effect on the gluten network than wheat starch, although the authors emphasized that simplified starch–gluten models cannot fully reproduce the behavior of complete wheat dough. The much stronger weakening produced by PF than by PS in the present study therefore suggests that gluten dilution alone was not the dominant factor; the high hydration capacity and structural characteristics of the processed potato matrix appear to have intensified competition for water during dough development.

The farinograph quality number reflected these differences in mixing tolerance. The control had an FQN of 146 mm, while PS10 remained comparable at 151 mm. In contrast, the FQN decreased to 112 and 101.5 mm in PG10 and PG20 and to only 30.5 and 33.0 in PF10 and PF20. This ranking closely parallels the changes in dough stability and softening and identifies PF as the ingredient causing the greatest loss of resistance to mechanical mixing.

### 3.4. Chemical Composition of Raw Materials and Pasta

The chemical composition of the three potato-derived ingredients differed significantly (*p* < 0.05), and these differences were reflected in the corresponding pasta formulations (Table 5). The most pronounced compositional contrast was observed for PS, which contained only 0.48% d.b. protein, 0.27% d.b. ash and 3.38% d.b. TDF, compared with 14.55, 0.74 and 6.37% d.b., respectively, in durum wheat semolina. In contrast to PS, PF and PG contained higher levels of non-starch components, with 8.85–9.21% d.b. protein, 2.61–2.94% d.b. ash, and 8.58–9.63% d.b. TDF. The latter was distributed differently between the two ingredients: PF contained relatively balanced IDF and SDF fractions (5.16 and 4.47% d.b., respectively), whereas PG was characterized predominantly by IDF (7.44% d.b.) and contained only 1.14% d.b. SDF. These compositional differences further support the distinction between the starch-rich PS ingredient and the more compositionally complex PF and PG matrices.

The composition of processed potato ingredients is strongly dependent on degree of processing and physical characteristics. Buzera et al. [12] demonstrated that blanching, boiling, oven drying and freeze-drying produced potato flours with significantly different protein, ash, fiber and carbohydrate contents; protein, for example, ranged from 6.41 to 10.17%, while ash ranged from 3.50 to 4.33%. Such processing-dependent variability is relevant when comparing potato ingredients across studies and may partly explain why materials described generically as potato flour, powder, flakes or grits can differ substantially in their nutritional composition and subsequent technological behavior.

Protein content was the pasta component most clearly affected by PS incorporation. Compared with 14.78% d.b. in the control, protein decreased to 13.55% d.b. in PS10 and further to 12.51% d.b. in PS20, with both formulations differing significantly (*p* < 0.05) from the control. This reduction is principally a dilution effect because PS contained almost no protein relative to semolina. The response was less pronounced for PF and PG, which contained substantially more protein than PS. PF pasta contained 13.92–13.86% d.b. protein, while PG10 retained 14.63% d.b. and PG20 contained 13.71% d.b. Therefore, although all three ingredients replaced the same proportion of semolina, their effects on protein concentration differed according to the protein content of the substituting material.

A similarly limited change in pasta protein has been reported when potato-derived materials with appreciable endogenous protein are incorporated at moderate levels. Gumul et al. [37], for example, used freeze-dried red potato pulp containing 11.32% protein and found essentially unchanged pasta protein at 10 and 20% substitution relative to the control, with only minor differences at higher incorporation. In contrast, stronger reductions are expected when wheat is replaced by predominantly starch-based ingredients because the substitution directly lowers the concentration of gluten-forming proteins.

Ash content showed the opposite pattern. PS incorporation did not produce a consistent increase relative to the control (0.81% d.b.), whereas PF and PG progressively increased ash to 1.35 and 1.26% d.b., respectively, at 20% substitution. This response corresponds directly to the greater mineral fraction of the raw PF and PG ingredients, which contained 2.94 and 2.61% d.b. as compared with only 0.27% d.b. in PS. Similar transfer of the mineral fraction from potato-derived ingredients to pasta has been reported by Gumul et al. [37], whose red potato pulp contained 4.78% ash and progressively increased pasta ash as the substitution level increased. Potato flour studies have likewise reported ash contents substantially higher than those typical of refined wheat flour, although the magnitude varies with potato cultivar and processing treatment [12].

The effect on dietary fiber was more dependent on ingredient type. PS significantly (*p* < 0.05) reduced TDF from 7.29% d.b. in the control to 5.16 and 5.18% d.b. in PS10 and PS20, respectively. This decrease was primarily associated with IDF, which fell from 4.49% d.b. in the control to approximately 2.60% d.b., while SDF remained statistically unchanged. In contrast, PF produced the numerically highest TDF contents, 8.17 and 8.19% d.b., with IDF reaching 5.61–5.68% d.b. PG resulted in TDF values of 6.94 and 7.51% d.b., with the higher substitution increasing the IDF fraction to 5.00% d.b. Importantly, however, neither PF nor PG produced a statistically significant increase in TDF relative to the control, whereas the reduction caused by PS was significant.

This response is consistent with the relatively moderate differences in TDF between semolina (6.37% d.b.) and the PF and PG ingredients (9.63 and 8.58% d.b., respectively). At substitution levels of only 10–20%, the resulting increase in the fiber contribution of the formulation would therefore be expected to remain limited. This contrasts with pasta fortified using highly concentrated potato fiber fractions. Namir et al. [25], for example, incorporated a potato-peel-derived fraction containing approximately 83% TDF and consequently obtained several-fold increases in pasta TDF even at relatively low inclusion levels.

Fat content remained low in all pasta formulations and did not differ significantly (*p* > 0.05) among samples, ranging from 0.13 to 0.27% d.b. This limited response was expected because all three potato materials were themselves low-fat ingredients. Moisture content of the dried pasta remained within a relatively narrow range of 8.67–9.83%. PF and PG showed slightly lower residual moisture than the control, with the lowest value observed for PG20, whereas PS20 had the highest moisture content. Given the significant differences in hydration behavior observed during farinograph analysis, these relatively small differences after drying suggest that the drying treatment largely reduced the initial formulation-dependent differences in water uptake, although the final moisture retained by the matrix remained ingredient-dependent.

Carbohydrate content represented the major macronutrient fraction in all pasta formulations and varied only slightly among samples, from 77.67% in the control to 82.04% in PS20. The higher numerical values observed for PS10 and PS20 (81.08 and 82.04%, respectively) are consistent with the predominantly starch-based composition of PS, whereas pasta containing PF and PG remained closer to the control, with values of 77.81–78.15% and 78.17–78.53%, respectively. This behavior reflects the greater contribution of protein, ash and dietary fiber from PF and PG compared with the starch-rich PS ingredient. Similar compositional shifts have been reported in wheat–potato systems, where increasing the proportion of potato-derived ingredients modifies the relative contribution of starch, protein, ash and fiber according to the composition of the added material [19].

Despite these compositional differences, the calculated energy value remained relatively stable, ranging from 384.28 to 390.97 kcal/100 g. PS-containing pasta showed the highest numerical values, reaching 390.72 and 390.97 kcal/100 g at 10 and 20% substitution, respectively, whereas PF formulations showed the lowest values (384.49 and 384.28 kcal/100 g). PG pasta remained intermediate, at 387.38 and 386.24 kcal/100 g, compared with 386.68 kcal/100 g for the control. The narrow range is expected because starch and protein contribute similar metabolizable energy on a weight basis, while the relatively low-fat contents of all formulations limited the contribution of the most energy-dense macronutrient. Consequently, replacement of semolina with the potato-derived ingredients modified the macronutrient distribution without materially altering the overall calculated energy density of the pasta.

### 3.5. Cooking Quality

Cooking behavior was strongly dependent on the type of potato-derived ingredient, with the clearest differences observed for MCT, WII and CL (Table 6). The control required 8.75 min to reach the cooking endpoint. PS caused only a limited reduction, to 8.25 and 8.00 min at 10 and 20% substitution, respectively, and these values did not differ significantly (*p* > 0.05) from the control. In contrast, incorporation of PF significantly (*p* < 0.05) shortened cooking time to 6.50 min at 10% and 6.00 min at 20%, while PG10 also significantly (*p* < 0.05) reduced it to 7.00 min. PG20 showed an intermediate value of 7.50 min that was not significantly (*p* > 0.05) different from the control. Thus, PF exerted the strongest effect on cooking time, whereas the response to PG was not concentration-dependent.

The shorter cooking time of the PF formulations is consistent with the pronounced hydration behavior observed during farinograph analysis and with the degree of processing and physical characteristics of potato flakes. Commercial potato flakes are produced by cooking, mashing, and drying potato tissue, during which the starch becomes gelatinized (Table 2) and acquires cold-water swelling capacity [11]. Consequently, part of the starch in PF is already structurally modified before incorporation into pasta and may require less additional hydration and thermal disruption during cooking than native semolina starch. At the same time, the very low dough stability observed for PF indicates that the gluten–starch matrix was considerably weakened, which may facilitate water penetration into the pasta structure and accelerate disappearance of the uncooked core. A reduction in cooking time after potato incorporation has also been reported by Sharma et al. [22], who observed shorter MCT in durum pasta containing either potato flour or potato mash. Sharma et al. [21] similarly reported a progressive decrease in cooking time when potato flour was incorporated into pasta and related this response to differences in starch structure, water absorption and the resulting protein–starch matrix.

The relatively small change produced by PS agrees more closely with observations for pasta enriched with isolated starches. Kolarič et al. [24] found only a slight decrease in optimum cooking time (OCT) when durum semolina was partially replaced with sweet-potato starch and attributed the response principally to dilution of the gluten-forming fraction and faster hydration of the modified starch–protein matrix. The weaker effect of PS compared with PF therefore supports the view that differences in the structural and hydration characteristics of the potato ingredients, including those associated with degree of processing, contributed to the observed variation in cooking rate.

The WII showed a contrasting response. PS significantly (*p* < 0.05) increased the index from 2.40 in the control to 2.50 and 2.58 for PS10 and PS20, respectively. Conversely, PF reduced weight increase to 2.28 and 2.08, while PG10 also showed a lower value of 2.30; PG20, at 2.35, remained statistically comparable to the control. The greater weight gain of PS pasta is consistent with the high swelling capacity of potato starch during heating. Although PS reduced farinograph water absorption under non-heated mixing conditions, this does not contradict its greater water uptake during cooking, because heating promotes starch granule swelling, loss of crystalline order, and increased water uptake within the cooked matrix [10].

The lower cooking weight gain of PF is particularly noteworthy because PF showed the highest farinograph water absorption. These two observations describe different stages of hydration. The high farinograph value reflects the strong ability of the preprocessed flake matrix to bind water during dough formation, whereas the cooking weight increase represents further water uptake and retention during thermal treatment. Because pregelatinized potato-flake starch already possesses high cold-water swelling capacity, a substantial part of its hydration potential may be expressed before cooking [11]. Moreover, the weak and poorly stable dough formed with PF may be less capable of retaining swollen starch and absorbed water during boiling.

CL provided the clearest evidence of differences in pasta structural integrity. The control released 5.40% d.b. into the cooking water, and PS incorporation produced essentially unchanged values of 5.19 and 5.42% d.b. at 10 and 20%, respectively. In contrast, CL increased significantly (*p* < 0.05) with both PF and PG. PF10 and PF20 reached 6.28 and 7.99% d.b., respectively, whereas PG increased from 7.36% d.b. at 10% substitution to 9.33% d.b. at 20%, the highest value among all formulations.

Similar increases in cooking loss have been reported for wheat pasta enriched with potato peel fiber, although the values remained below 8% [25]. Kolarič et al. [24] also reported increased cooking loss following the incorporation of sweet potato starch, reaching approximately 8.6% at 10% substitution. The higher losses observed in PF and particularly PG pasta may be associated with disruption of the continuous gluten network by fiber particles and potato cellular fragments, as well as competition for water during dough formation. Differences in particle size and structural state of the potato materials may also have contributed to this response. PG had the largest mean particle size (336.89 µm) and the smallest fine fraction (<160 µm; 9.67%), and its starch was only partly gelatinized (76.4%), whereas PF particles were somewhat smaller (302.61 µm) but almost completely pregelatinized (98.04%), and PS consisted entirely of fine, native starch granules (Table 2). Coarse particles containing tuber cell-wall fragments are likely to hydrate less uniformly during the short mixing stage and to be less effectively embedded in the protein matrix, which may create local discontinuities from which solids can leach during cooking. A similar relationship was reported by Buzera et al. [17], who attributed the higher cooking loss of noodles containing oven-dried potato flour to its larger particle size and higher degree of starch gelatinization. These effects may weaken the protein matrix and reduce its capacity to retain gelatinized starch and other soluble components during cooking, thereby promoting their leaching into the cooking water. The cooking loss of PG20 (9.33%) exceeded the approximately 8% threshold commonly associated with good-quality pasta, although it remained below the 10% upper limit sometimes considered acceptable. This result suggests reduced structural integrity and potentially greater surface stickiness of the cooked pasta. Since sensory evaluation was beyond the scope of this study, which aimed to compare the technological and nutritional effects of the potato ingredients, the consumer acceptability of PG20 and of the harder and more adhesive PF-containing pasta should be verified by sensory testing. In contrast, the low CL values of PS pasta indicate that substitution with this starch-rich ingredient, at levels up to 20%, did not substantially compromise the retention of solids during cooking.

### 3.6. Texture Profile Analysis (TPA)

The texture of the cooked pasta was strongly influenced by the type of potato-derived ingredient, with the most pronounced changes observed for hardness, cohesiveness and springiness (Table 7). The control showed a hardness of 11.32 N, while PS10 remained statistically comparable at 5.54 N. Increasing PS to 20% significantly (*p* < 0.05) increased hardness to 24.65 N. Although PS10 and PS20 exhibited similar cooking losses and cooking times, the marked increase in hardness at the higher substitution level suggests that pasta hardness was affected not only by these parameters but also by changes in the structure of the pasta matrix and water availability in the cooked product. A higher proportion of potato starch may induce structural changes that increase the resistance of the pasta to compression. Much larger increases were observed for PF and PG, with PF10 and PF20 exhibiting the highest values (81.53 and 56.45 N, respectively). The high hardness of pasta containing PF, despite their shorter cooking time and higher cooking losses, may be attributed to the lower water uptake capacity of these formulations, potentially resulting in lower moisture content in the cooked pasta and greater resistance to compression. Interestingly, although the potato flakes themselves exhibited high WAI (Table 2), their incorporation into the pasta matrix substantially reduced the water uptake of the final product during cooking. Pasta containing 10% and 20% potato grits (PG10 and PG20) also exhibited relatively high hardness. This may be associated with changes in the pasta matrix structure and water distribution caused by the incorporation of potato grits.

The high hardness of PF-containing pasta should not, however, be interpreted as evidence of a stronger or more cohesive gluten network. In starch-rich cereal systems, hardness reflects the combined contribution of the protein matrix, starch swelling and gelatinization, and the development of starch–protein associations during cooking. Tao et al. [10] likewise reported that potato starch increased noodle hardness and proposed that gelatinized starch and starch–protein interactions can reinforce resistance to compression even while the gluten network itself becomes progressively disrupted. A related response was observed by Tao et al. [38], who found that potato powder increased noodle hardness despite the appearance of a looser and more discontinuous gluten network; the authors attributed this to the contribution of pregelatinized starch gel and starch–protein structures formed during processing. As no microstructural analyses (e.g., SEM or CLSM) were performed in the present study, the proposed changes in gluten-network continuity and starch–protein interactions are inferred indirectly from rheological, cooking and texture data and should be regarded as hypotheses.

This interpretation is particularly relevant to PF because potato flakes contain previously cooked and gelatinized starch. Their high water-binding capacity and altered starch structure can favor formation of a dense starch-rich phase during cooking, increasing the force required for initial compression. At the same time, competitive hydration of potato flakes has been shown to interfere with gluten development. In wheat–potato systems, potato flakes displayed significantly (*p* < 0.05) greater water absorption than either wheat starch or gluten, and this competition for water produced a discontinuous gluten network [16]. The combination of high hardness with poor structural recovery observed for PF in the present study therefore suggests a rigid but weakly cohesive cooked matrix, rather than reinforcement of the native semolina gluten structure.

The non-linear response of hardness to PF concentration supports this interpretation. Hardness decreased significantly (*p* < 0.05) from 81.53 N at 10% PF to 56.45 N at 20%, although both values remained far above the control. At moderate substitution, swollen or gelatinized potato starch may contribute to matrix filling and resistance to compression, whereas further replacement of semolina increasingly dilutes gluten and disrupts network continuity. A similar concentration-dependent balance has been reported in potato-enriched noodles. Buzera et al. [17] found that low levels of potato flour could maintain or increase hardness, whereas higher substitution significantly (*p* < 0.05)reduced it as gluten dilution and structural weakening became dominant.

Adhesiveness showed a similarly strong dependence on ingredient type. The control and PS10 exhibited no measurable adhesiveness, while PS20 remained very low at 0.06 N·s. In contrast, PF10 and PF20 reached 1.92 and 2.02 N·s, respectively, and PG20 showed an intermediate value of 1.12 N·s; PG10, at 0.42 N·s, was not significantly (*p* > 0.05) different from the control. The greater adhesiveness of PF is consistent with its high hydration capacity and the greater availability of gelatinized or damaged starch at the pasta surface. During cooking, disruption of the protein matrix can facilitate migration and leaching of starch toward the surface, increasing stickiness. This interpretation is consistent with the higher cooking losses observed for PF and PG and with previous studies linking weakened gluten continuity to increased surface starch and adhesiveness in potato-containing cereal products [24].

Cohesiveness and springiness index provided clearer evidence of the loss of structural integrity caused by PF and PG. Cohesiveness remained high and statistically unchanged in CON and PS10, at 0.96 and 0.97, respectively, but decreased to 0.80 in PS20. Much greater reductions occurred with PF, reaching 0.36 and 0.34 at 10 and 20% substitution. PG produced intermediate values of 0.58 and 0.40. Springiness index followed a similar pattern: CON, PS10 and PS20 remained statistically comparable at 0.99, 0.99 and 0.89, whereas PG10 decreased to 0.71 and PF10, PF20 and PG20 showed the lowest values, between 0.36 and 0.39.

These reductions are consistent with progressive disruption of the gluten network and replacement of an elastic protein-dominated structure by a more discontinuous starch-rich matrix. Cohesiveness reflects the ability of the cooked structure to withstand deformation without internal rupture, while springiness describes its ability to recover after compression. Likewise, Buzera et al. [17] showed that potato flour reduced both cohesiveness and springiness of cooked noodles, particularly at higher substitution levels, and related these changes to weakening of the starch–gluten network. Microstructural study of wheat noodles have further shown that springiness is closely associated with the continuity and branching characteristics of the gluten network, and that starch swelling behavior can substantially alter this network during cooking [39].

### 3.7. Color Analysis of Pasta

**Figure 1:**
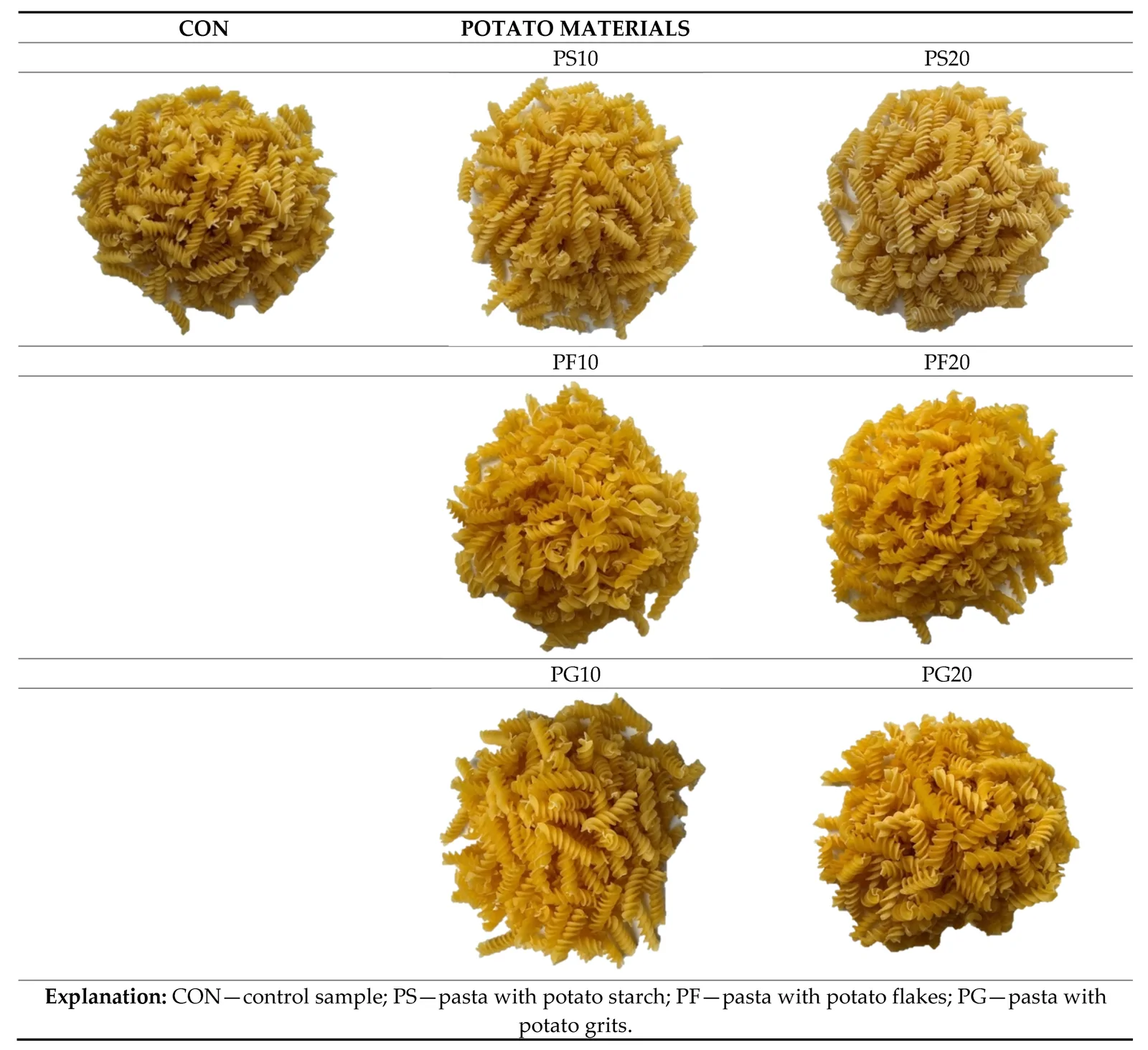
Photographs of the obtained pasta samples with potato-derived ingredients.

The three potato-derived ingredients differed significantly (*p* < 0.05) in their intrinsic color characteristics (Figure 1), which were reflected in the color characteristics of the corresponding dry pasta (Table 8).

Among the raw materials, PS showed the highest lightness (L* = 92.66) and whiteness index (*WI* = 92.21), whereas PG was the darkest material (L* = 79.49) and exhibited the greatest yellowness (b* = 22.29; *YI* = 40.08). PF showed intermediate values, with an L* of 83.76 and b* of 14.41, compared with 87.13 and 19.63, respectively, for semolina. The significant differences (*p* < 0.05) among the raw materials indicate that each potato ingredient provided a distinct initial chromatic contribution to the pasta formulations.

The effect on pasta lightness differed according to ingredient type and substitution level. The control showed an L* value of 56.31. PS10 remained statistically comparable to the control (54.53), whereas increasing PS to 20% significantly (*p* < 0.05) increased lightness (*p* < 0.05) to 59.79, consistent with the particularly high L* of the raw PS ingredient. In contrast, PF decreased L* to 52.81 and 51.86 at 10 and 20% substitution, respectively, both significantly (*p* < 0.05) below the control. PG10 also produced a significantly (*p* < 0.05) darker pasta (L* = 53.49), whereas PG20 (56.75) did not differ significantly (*p* >0.05) from the control. *WI* followed essentially the same pattern, reaching its highest value in PS20 (53.52) and its lowest in PF20 (44.87).

The decrease in lightness observed particularly with PF agrees with previous studies on wheat–potato products. Sharma et al. [22] reported a progressive reduction in L* when potato flour or potato mash was incorporated into durum wheat pasta, with the decrease being more pronounced for potato flour. Bao et al. [15] similarly observed that increasing potato flour substitution progressively reduced the lightness of wheat noodles, irrespective of whether the potato flour was ethanol-, freeze- or oven-dried. Importantly, however, the magnitude of the response differed according to the drying method, demonstrating that potato processing history itself influences the optical properties subsequently expressed in the cereal product. Moreover, Buzera et al. [17] also reported lower L* values in potato-enriched noodles and showed that the method used to process the potato flour affected the resulting noodle color.

Changes in the chromatic coordinates were more moderate. The a* value of the control was 3.03, whereas both PS formulations showed significantly lower values (*p* < 0.05) (2.58 and 2.45). PF and PG generally remained statistically comparable to the control, with PG20 showing the highest a* value (3.25). For b*, only PF20 differed significantly (*p* < 0.05) from the control, increasing from 23.81 to 26.69, although numerically higher values were also obtained for PF10, PG10 and PG20. A similar pattern was evident for *YI*: PF20 showed the highest value (73.53), while PG10 also exhibited significantly greater yellowness (*p* < 0.05) than the control.

The total color difference (ΔE*) further highlighted the ingredient-specific response. Relative to the control, PF20 showed the greatest color difference (ΔE* = 5.42), followed by PF10 (4.21), PS20 (3.61), PG10 (3.41), PS10 (2.55) and PG20 (2.25), with all supplemented formulations differing significantly from each other (*p* < 0.05). The particularly large change in PF20 is consistent with its simultaneous reduction in L* and *WI* and increase in b* and *YI*. By contrast, the relatively low ΔE* of PG20 despite the darker and more yellow appearance of the raw PG material indicates that the final pasta color was not a simple linear reflection of raw-material color. Previous studies likewise indicate that the color of wheat–potato products depend on the processing method and the physicochemical characteristics of the potato ingredient, in addition to its incorporation level [15,17].

### 3.8. Digestible and Resistant Starch Content in Pasta

Post-cooking storage affected both resistant starch (RS) and digestible starch, but the response depended on storage temperature and the type of potato ingredient (Table 9). After 24 h at 4 °C, RS was not detected in the control and in all PS- and PF-containing samples. Detectable RS was found only in PG10 and PG20, at 0.56 and 0.69% d.b., respectively, with PG20 showing a significantly (*p* < 0.05) higher value than PG10 (*p* < 0.05). In contrast, after storage at −18 °C for 24 h, RS was detected in all formulations and ranged from 0.58% d.b. in PF10 to 3.18% d.b. in PG20. Relative to the frozen control (0.73% d.b.), RS was significantly higher (*p* < 0.05) in PS10 (1.21% d.b.), PS20 (2.53% d.b.), PG10 (1.84% d.b.), and PG20 (3.18% d.b.). PF20 (0.89% d.b.) did not differ significantly (*p* > 0.05) from the control, whereas PF10 showed a significantly lower value (*p* < 0.05). PG20 exhibited the highest RS content, followed by PS20.

The limited RS formation observed after refrigerated storage is consistent with evidence showing that short-term refrigeration does not necessarily produce a large increase in RS in dry pasta. Cañas et al. [29] found that refrigeration at 4 °C for 24 h had relatively little effect on the starch digestibility of boiled dry pasta, whereas the response was more evident in fresh pasta. Sissons et al. [30] similarly reported only a minor increase in RS after refrigerated storage of cooked spaghetti and found no corresponding reduction in the overall extent of starch digestion. In contrast, Kolarič et al. [24] observed increases of approximately 20–27% in RS after sweet-potato-starch-enriched pasta was stored at 5–8 °C for 24 h. The different responses may reflect differences in starch source, initial RS content, degree of gelatinization, formulation and the organization of the starch–protein matrix.

The higher RS contents obtained after freezing are consistent with the formation of starch structures with reduced enzymatic susceptibility during post-cooking storage. Following gelatinization, amylose and, over longer periods, amylopectin chains can reassociate through hydrogen bonding and form more ordered structures that are less accessible to amylolytic enzymes. Low-temperature treatments may additionally modify water distribution within the matrix; ice formation can concentrate starch chains in the unfrozen phase and reduce intermolecular distances, potentially favoring molecular associations during freezing or subsequent temperature changes. Feng et al. [5] discussed these mechanisms for low-temperature and freeze–thaw treatments, while emphasizing that the development of enzyme-resistant retrograded structures depends on temperature history, moisture and starch characteristics.

The greater RS accumulation observed following storage at −18 °C compared with 4 °C in the present formulations should nevertheless be considered specific to this pasta system rather than a universal effect of freezing. Tian et al. [31] reported significant RS formation in cooked noodles stored at −18, 4 and 25 °C, but the greatest RS levels occurred at 25 °C, indicating that sufficient molecular mobility can be as important as low-temperature nucleation. Similarly, Yi et al. [32] found that 4 °C storage was more effective than −18 °C in reducing starch digestibility in cooked rice. These contrasting findings emphasize that storage temperature interacts with moisture content, starch molecular structure and food-matrix organization rather than determining retrogradation independently. Because only a single 24 h storage period was applied here, longer storage or repeated freeze–thaw cycles could further increase RS, as amylopectin retrogradation continues over several days and each thawing step increases chain mobility before refreezing again concentrates starch in the unfrozen phase [5,31]; however, repeated freeze–thaw cycles have also been shown to reduce the water-holding capacity, chewiness and sensory scores of frozen cooked noodles [40], so any additional gain in RS would need to be balanced against product quality.

Ingredient type also influenced the response to freezing. The concentration-dependent increase observed for PS, from 1.21% d.b. in PS10 to 2.53% d.b. in PS20, is consistent with the starch-rich composition of PS. PG showed an even stronger response, reaching 1.84 and 3.18% d.b. at 10 and 20% substitution, respectively. Conversely, PF produced only 0.58–0.89% d.b. RS despite its high hydration capacity and thermally processed starch matrix. This contrast suggests that the capacity to generate enzyme-resistant starch after cooking cannot be predicted simply from water absorption or the presence of potato starch. Differences in previous thermal treatment, extent of starch gelatinization or damage, cellular organization and water distribution could influence the mobility and reassociation of starch chains during storage. Protein–starch interactions may also contribute, since proteins can either promote or restrict retrogradation depending on their interactions with starch and water [28]. These differences may also reflect the state of the amylose and amylopectin fractions in each ingredient. After gelatinization, amylose chains reassociate rapidly into thermally stable double helices that largely determine short-term RS3 formation, whereas recrystallization of the outer amylopectin chains is slower and yields less stable crystallites [5,41]. In PS, the almost entirely native starch (Table 2) was fully gelatinized during cooking, releasing a large pool of amylose available for reassociation, which is consistent with its concentration-dependent RS increase. In PF, the starch had already been gelatinized and partly retrograded during drum-drying (98.04%), and part of the amylose may have been leached, complexed with lipids or entrapped within intact cells, limiting its mobility during storage [11,14], while the partly gelatinized starch of PG (76.4%) remained embedded in cell-wall material that may locally restrict water availability and favor chain association. Phosphate monoesters bound to potato amylopectin, which increase hydration and may hinder close chain packing, could further modulate retrogradation [9,35]. Because amylose content, amylopectin chain-length distribution and starch crystallinity were not determined, these interpretations remain hypotheses requiring confirmation, for example by XRD and DSC.

Freezing was also accompanied by a significant reduction (*p* < 0.05) in digestible starch for every formulation. Values after refrigeration ranged from 60.17 to 68.16% d.b., whereas frozen samples ranged from 62.98 to 66.50% d.b. The lowest values after freezing were recorded for PG20 (62.98% d.b.), which was significantly lower (*p* < 0.05) than in freshly cooked pasta (67.64% d.b.) as well as in pasta cooled at 4 °C for 24 h (65.93% d.b.). From a nutritional perspective, the absolute RS contents remain moderate: assuming a portion of 80–100 g of dry pasta (approximately 90% dry matter), PG20 stored at −18 °C would provide approximately 2.3–2.9 g of RS, roughly half of the estimated mean habitual RS intake of 4.9 g per day reported for the US population [42]. At the same time, RS accounted for only about 5% of total starch in PG20 (Table 9), well below the level of at least 14% RS in total starch associated with the EU-authorised health claim on post-prandial glycaemic responses [43], so the observed increase should be regarded as a moderate nutritional improvement rather than one sufficient to support a health claim.

## 4. Conclusions

The incorporation of potato-derived ingredients modified the technological, compositional and starch-digestibility characteristics of durum wheat pasta, with the response depending strongly on ingredient type and substitution level. PS produced the least disruption to dough stability and cooking quality and promoted greater water uptake during cooking, whereas PF showed the highest hydration requirement during mixing and substantially weakened dough stability, resulting in a firmer but less cohesive and more adhesive cooked structure. PG produced an intermediate rheological response but increased cooking loss, particularly at the higher substitution level. These differences indicate that potato-derived ingredients should not be considered technologically equivalent, as their composition and processing history influence their interaction with the semolina gluten–starch matrix.

Post-cooking storage was also a major determinant of starch susceptibility to enzymatic digestion. Refrigerated storage for 24 h resulted in little detectable RS formation in most formulations, whereas storage at −18 °C increased RS in the most samples, with the greatest accumulation observed in PG20 and PS20. The simultaneous decrease in the enzyme-susceptible starch fraction is consistent with the development of structures less accessible to enzymatic hydrolysis during frozen storage. However, because starch molecular organization and crystallinity were not directly characterized, the underlying structural mechanisms require further confirmation.

Overall, the results demonstrate that ingredient processing state and post-cooking storage conditions act together to determine the balance between pasta technological quality and resistant starch formation. Among the tested formulations, PS better preserved conventional pasta quality, whereas PG showed greater potential for enhancing RS formation after frozen storage, although this was accompanied by deterioration in some cooking and textural attributes. These findings provide a basis for selecting potato-derived ingredients according to the desired technological and nutritional characteristics of durum wheat pasta. However, because the RS levels achieved remain moderate and sensory acceptability, microstructure and batch-to-batch reproducibility were not evaluated, these findings should be confirmed before practical application, particularly for PG20, in which the highest RS content was accompanied by the highest cooking loss.

## Figures and Tables

**Table 1:** Model of experiment and pasta processing parameters.

Samples	Pasta Formula				Production Parameters			
	Semolina	Potato Materials			Pressure of Mixing	Pressure of Extrusion	Extruder Capacity	Barrel Temp.
		Potato Starch	Potato Flakes	Potato Grits				
	[%]				[MPa]	[MPa]	[kg h^−1^]	[°C]
CON	100	-	-	-	0.08	18	24.60 ^c^ ± 0.34	28.8
PS10	90	10	-	-	0.08	15.5	28.26 ^b^ ± 0.93	29.0
PS20	80	20	-	-	0.08	18	30.48 ^a^ ± 0.34	29.7
PF10	90	-	10	-	0.08	12	25.26 ^c^ ± 0.42	28.5
PF20	80	-	20	-	0.08	14	25.74 ^c^ ± 0.25	29.2
PG10	90	-	-	10	0.08	14	25.14 ^c^ ± 0.08	28.7
PG20	80	-	-	20	0.08	16.5	26.16 ^c^ ± 0.34	29.4

Explanation: CON—control sample; PS—pasta with potato flour; PF—pasta with potato flakes; PG—pasta with potato grits. Means (*n* = 2) with different letters (a–c) in the same column are significantly different (Tukey test; *p* < 0.05).

**Table 2:** Physicochemical and functional properties of raw materials.

Raw Materials	Mean Particle Size (µm)	Fine Fraction (<160 µm) (%)	Degree of Starch Gelatinisation (%)	WAI (%)	WSI (%)	Total Starch Content (% d.b.)	RS Content (% d.b.)
PS	63.32 ^d^ ± 0.01	100.00 ^a^ ± 0.00	5.09 ^d^ ± 0.73	176.58 ^d^ ± 5.98	1.15 ^d^ ± 0.05	85.54 ^a^ ± 1.21	22.48 ^a^ ± 0.71
PG	336.89 ^a^ ± 4.82	9.67 ^d^ ± 1.26	76.4 ^b^ ± 0.31	569.51 ^b^ ±12.43	11.74 ^b^ ± 0.12	76.73 ^b^ ± 0.92	6.90 ^b^ ± 0.21
PF	302.61 ^b^ ± 2.68	15.57 ^c^ ± 0.69	98.04 ^a^ ± 0.98	712.14 ^a^ ± 27.23	30.79 ^a^ ± 3.89	72.75 ^c^ ± 0.84	2.05 ^c^ ± 0.09
DWS	155.77 ^c^ ± 1.89	52.13 ^b^ ± 2.86	18.4 ^c^ ± 0.19	200.17 ^c^ ± 2.11	6.33 ^c^ ± 0.27	69.10 ^d^ ± 0.53	2.39 ^c^ ± 0.42

Explanation: PS—potato starch; PG—potato grits; PF—potato flakes; DWS—durum wheat semolina; WAI—water absorption index; WSI—water solubility index, RS—resistant starch. Data are presented as mean (*n* = 3) ± standard deviation; means in the same column with different letters (a–d) are significantly different (Tukey test; *p* < 0.05).

**Table 3:** Pasting characteristics of raw materials.

Raw Materials	Peak Viscosity [cP]	Peak Time [min]	Pasting Temp [oC]	Holding Strength	Breakdown	Final Viscosity	Setback
**PS**	8350.3 ^a^ ± 143.6	12.4 ^b^ ± 0.1	55.5 ^a^ ± 5.7	1694.0 ^a^ ± 115.1	6656.7 ^a^ ± 114.7	2706.3 ^a^ ± 94.1	1012.3 ^a^ ± 37.5
**PG**	1426.1 ^c^ ± 42.7	25.7 ^a^ ± 0.2	56.2 ^a^ ± 4.2	1243.0 ^b^ ± 99.5	182.6 ^c^ ± 3.6	1570.0 ^b^ ± 45.1	326.9 ^d^ ± 10.2
**PF**	2865.0 ^b^ ± 85.1	1.03 ^c^ ± 0.01	54.4 ^a^ ±.3.9	792.5 ^c^ ± 25.2	2073 ^b^ ± 41	1222 ^d^ ± 39	429.3 ^c^ ± 11.5
**DWS**	888.7 ^d^ ± 17.6	24.2 ^a^ ± 0.3	56.3 ^a^ ± 2.1	719.2 ^c^ ± 19.8	169.5 ^c^ ± 2.8	1381 ^c^ ± 35	661.7 ^b^ ± 14.6

Explanation: PS—potato starch; PG—potato grits; PF—potato flakes; DWS—durum wheat semolina. Data are presented as mean (*n* = 3) ± standard deviation; means in the same column with different letters (a–d) are significantly different (Tukey test; *p* < 0.05).

**Table 4:** Farinograph characteristics of mixtures.

Samples	Development Time	Water Absorption	Dough Stability	Degree of Softening (After 12 min)	Farinograph Quality Number
	min	[%]	min	FU	mm
**CON**	1.83 ^b^ ± 0.05	61.45 ^e^ ± 0.07	15.37 ^a^ ± 0.01	25.00 ^e^ ± 5.66	146.02 ^b^ ± 0.04
**PS10**	1.87 ^b^ ± 0.01	59.35 ^f^ ± 0.07	14.54 ^b^ ± 0.01	28.00 ^d^ ± 1.41	151.03 ^a^ ± 0.06
**PS20**	1.85 ^b^ ± 0.02	57.30 ^g^ ± 0.14	>20 *	23.50 ^f^ ± 3.54	>200 *
**PF10**	2.27 ^b^ ± 0.05	76.30 ^c^ ± 0.42	1.25 ^e^ ± 0.02	212.00 ^a^ ± 38.18	30.50 ^f^ ± 2.12
**PF20**	2.52 ^b^ ± 0.05	85.95 ^a^ ± 0.21	1.00 ^f^ ± 0.07	284.05 ± 25.67	33.00 ^e^ ± 0.07
**PG10**	6.43 ^a^ ± 0.67	71.60 ^d^ ± 0.14	8.44 ^c^ ± 0.08	88.50 ^c^ ± 4.95	112.00 ^c^ ± 1.41
**PG20**	7.21 ^a^ ± 0.08	81.30 ^b^ ± 0.01	5.13 ^d^ ± 0.09	128.50 ^b^ ± 0.71	101.50 ^d^ ± 0.71

* The measurement was stopped after 20 min, as dough consistency remained unchanged. Explanation: CON—control sample (100% semolina durum pasta); PS—pasta with potato starch; PF—pasta with potato flakes; PG—pasta with potato grits. Data are presented as mean (*n* = 2) ± standard deviation; means in the same column with different letters (a–g) are significantly different (Tukey test; *p* < 0.05).

**Table 5:** Chemical compositions of raw materials and pasta samples.

Samples	Moisture Content	Ash Content	Protein	Fat	IDF	SDF	TDF	Carbohydrate	Energy
	[%]	[% d.b.]							[kcal/100 g d.b.]
Raw Materials									
DWS	13.87 ^A^ ± 0.04	0.74 ^C^ ± 0	14.55 ^A^ ± 0.04	1.18 ^A^ ± 0.02	4.24 ^C^ ± 0.17	2.13 ^B^ ± 0.22	6.37 ^B^ ± 0.45	77.16	390.20
PS	11.95 ^B^ ± 0.06	0.27 ^D^ ± 0.01	0.48 ^D^ ± 0.02	0.10 ^C^ ± 0.01	2.10 ^D^ ± 0.17	1.28 ^C^ ± 0.41	3.38 ^C^ ± 0.58	95.77	392.66
PF	6.76 ^D^ ± 0.03	2.94 ^A^ ± 0.02	8.85 ^C^ ± 0.12	0.46 ^B^ ± 0.02	5.16 ^B^ ± 0.37	4.47 ^A^ ± 0.72	9.63 ^A^ ± 1.09	78.12	371.28
PG	8.41 ^C^ ± 0.02	2.61 ^B^ ± 0.01	9.21 ^B^ ± 0.05	0.12 ^C^ ± 0.03	7.44 ^A^ ± 0.01	1.14 ^C^ ± 0.48	8.58 ^A^ ± 0.48	79.48	373.00
Pasta samples									
CON	9.57 ^ab^ ± 0.20	0.81 ^c^ ± 0.24	14.78 ^a^ ± 0.04	0.25 ^a^ ± 0.04	4.49 ^ab^ ± 0.13	2.81 ^a^ ± 0.16	7.29 ^a^ ± 0.04	76.87	383.43
PS10	9.28 ^bc^ ± 0.18	0.90 ^bc^ ± 0.01	13.55 ^e^ ± 0.03	0.21 ^a^ ± 0.04	2.59 ^b^ ± 0.42	2.58 ^a^ ± 0.03	5.16 ^b^ ± 0.44	80.18	387.13
PS20	9.83 ^a^ ± 0.14	0.81 ^c^ ± 0.01	12.51 ^f^ ± 0.04	0.27 ^a^ ± 0.04	2.60 ^b^ ± 0.79	2.58 ^a^ ± 0.08	5.18 ^b^ ± 0.87	81.23	387.75
PF10	9.04 ^cd^ ± 0.15	1.17 ^ab^ ± 0.03	13.92 ^c^ ± 0.06	0.16 ^a^ ± 0.03	5.61 ^a^ ± 0.77	2.56 ^a^ ± 0.37	8.17 ^a^ ± 0.40	76.58	379.78
PF20	9.02 ^cd^ ± 0.03	1.35 ^a^ ± 0.04	13.86 ^c^ ± 0.03	0.13 ^a^ ± 0.04	5.68 ^a^ ± 0.50	2.51 ^a^ ± 0.01	8.19 ^a^ ± 0.51	76.47	378.87
PG10	8.94 ^cd^ ± 0.14	1.11 ^ab^ ± 0.05	14.63 ^b^ ± 0.03	0.25 ^a^ ± 0.09	4.51 ^ab^ ± 0.16	2.43 ^a^ ± 0.37	6.94 ^ab^ ± 0.53	77.07	382.93
PG20	8.67 ^d^ ± 0.06	1.26 ^a^ ± 0.01	13.71 ^d^ ± 0.01	0.25 ^a^ ± 0.03	5.00 ^a^ ± 0.14	2.51 ^a^ ± 0.01	7.51 ^a^ ± 0.13	77.27	381.19

Explanation: IDF—insoluble dietary fiber; SDF—soluble dietary fiber; TDF—total dietary fiber; CON—control sample; PS—pasta with potato starch; PF—pasta with potato flakes; PG—pasta with potato grits. Data are presented as mean (*n* = 2) ± standard deviation. Data values of each parameter with different superscript letters (a–f) in the columns are significantly different (Tukey test, *p* < 0.05). Values for raw materials marked with different uppercase superscript letters (A–D) indicate statistically significant differences (Tukey’s test, *p* < 0.05), with pasta sample results excluded from this comparison.

**Table 6:** Cooking quality of pasta samples.

Samples	Minimum Cooking Time (MCT)	Weight Increase Index (WII)	Cooking Loss (CL)
	[min]		[% d.b.]
**CON**	8.75 ^a^ ± 0.35	2.40 ^b^ ± 0.01	5.40 ^e^ ± 0.23
**PS10**	8.25 ^ab^ ± 0.35	2.50 ^a^ ± 0.01	5.19 ^e^ ± 0.35
**PS20**	8.00 ^ab^ ± 0.00	2.58 ^a^ ± 0.03	5.42 ^e^ ± 0.23
**PF10**	6.50 ^cd^ ± 0.71	2.28 ^c^ ± 0.02	6.28 ^d^ ± 0.24
**PF20**	6.00 ^d^ ± 0.00	2.08 ^d^ ± 0.04	7.99 ^b^ ± 0.34
**PG10**	7.00 ^bcd^ ± 0.00	2.30 ^c^ ± 0.03	7.36 ^c^ ± 0.45
**PG20**	7.50 ^abc^ ± 0.00	2.35 ^bc^ ± 0.01	9.33 ^a^ ± 0.21

Explanation: CON—control sample (100% semolina durum pasta); PS—pasta with potato starch; PF—pasta with potato flakes; PG—pasta with potato grits. Data are presented as mean (*n* = 3) ± standard deviation; means in the same column with different letters (a–e) are significantly different (Tukey test; *p* < 0.05).

**Table 7:** Texture profile analysis (TPA) parameters of pasta samples at their optimal cooking time (OCT).

Samples	Hardness	Adhesiveness	Cohesiveness	Springiness Index
	N	N·s		
**CON**	11.32 ^e^ ± 4.78	n.d.	0.96 ^a^ ± 0.02	0.99 ^a^ ± 0.02
**PS10**	5.54 ^e^ ± 2.83	n.d.	0.97 ^a^ ± 0.03	0.99 ^a^ ± 0.01
**PS20**	24.65 ^d^ ± 4.53	0.06 ^c^ ± 0.09	0.80 ^b^ ± 0.18	0.89 ^a^ ± 0.12
**PF10**	81.53 ^a^ ± 9.95	1.92 ^a^ ± 0.48	0.36 ^d^ ± 0.05	0.39 ^c^ ± 0.12
**PF20**	56.45 ^b^ ± 4.43	2.02 ^a^ ± 0.90	0.34 ^d^ ± 0.04	0.36 ^c^ ± 0.05
**PG10**	44.97 ^c^ ± 3.13	0.42 ^bc^ ± 0.26	0.58 ^c^ ± 0.10	0.71 ^b^ ± 0.05
**PG20**	46.66 ^bc^ ± 6.27	1.12 ^ab^ ± 0.70	0.40 ^cd^ ± 0.10	0.38 ^c^ ± 0.07

Explanation: CON—control sample (100% semolina durum pasta); PS—pasta with potato starch; PF—pasta with potato flakes; PG—pasta with potato grits. Data are presented as mean (*n* = 5) ± standard deviation; means in the same column with different letters (a–e) are significantly different (Tukey test; *p* < 0.05); n.d.—not detected.

**Table 8:** Color parameters of dry pasta with potato material samples.

Samples	L*	a*	b*	∆E*	*YI*	*WI*
Raw materials						
Semolina	87.13 ^B^ ± 0.08	0.90 ^A^ ± 0.03	19.63 ^B^ ± 0.11	-	32.19 ^B^ ± 0.20	76.51 ^C^ ± 0.13
PS	92.66 ^A^ ± 0.21	−0.11 ^B^ ± 0.12	2.54 ^D^ ± 0.69	-	3.91 ^D^ ± 1.07	92.21 ^A^ ± 0.44
PF	83.76 ^C^ ± 1.09	−1.29 ^C^ ± 0.06	14.41 ^C^ ± 0.47	-	24.57 ^C^ ± 0.59	78.23 ^B^ ± 0.61
PG	79.49 ^D^ ± 1.03	0.85 ^A^ ± 0.11	22.29 ^A^ ± 0.70	-	40.08 ^A^ ± 1.60	69.69 ^D^ ± 1.06
Dry pasta						
CON	56.31 ^b^ ± 1.55	3.03 ^ab^ ± 0.42	23.81 ^b^ ± 2.81	-	60.45 ^cd^ ± 7.23	50.09 ^b^ ± 1.89
PS10	54.53 ^bc^ ± 2.16	2.58 ^c^ ± 0.14	24.16 ^ab^ ± 1.63	2.55 ^e^ ± 1.99	63.28 ^bc^ ± 3.39	48.41 ^bc^ ± 1.58
PS20	59.79 ^a^ ± 1.62	2.45 ^c^ ± 0.17	23.16 ^b^ ± 0.52	3.61 ^c^ ± 1.64	55.40 ^d^ ± 2.44	53.52 ^a^ ± 1.57
PF10	52.81 ^cd^ ± 0.91	2.72 ^bc^ ± 0.38	24.65 ^ab^ ± 2.33	4.21 ^b^ ± 1.00	66.68 ^bc^ ± 6.26	46.65 ^cd^ ± 1.25
PF20	51.86 ^d^ ± 1.04	2.84 ^abc^ ± 0.22	26.69 ^a^ ± 0.96	5.42 ^a^ ± 0.81	73.53 ^a^ ± 2.36	44.87 ^d^ ± 0.82
PG10	53.49 ^cd^ ± 1.16	3.07 ^ab^ ± 0.25	25.31 ^ab^ ± 0.89	3.41 ^d^ ± 0.76	67.61 ^ab^ ± 1.73	46.94 ^cd^ ± 0.79
PG20	56.75 ^b^ ± 1.26	3.25 ^a^ ± 0.33	25.56 ^ab^ ± 0.85	2.25 ^e^ ± 0.64	64.38 ^bc^ ± 2.90	49.65 ^b^ ± 1.31

Explanation: CIE–lab color scale, ∆E*–the total color difference, *YI*–the yellowness index, *WI*–the whiteness index. Mean values (*n* = 10) of each parameter with different superscript letters (a–e) in the columns are significantly different (Tukey test, *p* < 0.05). Values for raw materials marked with different uppercase superscript letters (A–D) indicate statistically significant differences (Tukey’s test, *p* < 0.05), with pasta sample results excluded from this comparison.CON—control sample; PS—potato starch; PF— potato flakes; PG— potato grits.

**Table 9:** Digestible and resistant starch content in pasta after cooling and freezing treatments.

Samples	Pasta After Cooking		Pasta After Cooking and Cooling (4 °C)		Pasta After Cooking and Freezing (−18° C)	
	Resistant Starch	Digestible Starch	Resistant Starch	Digestible Starch	Resistant Starch	Digestible Starch
	[% d.b.]		[% d.b.]		[% d.b.]	
CON	N.D.	68.04 ^abA^ ± 0.34	N.D.	66.68 ^bB^ ± 0.26	0.73 ^e^ ± 0.04	66.50 ^aB^ ± 0.13
PS10	N.D.	66.47 ^cA^ ± 0.27	N.D.	65.02 ^cB^ ± 0.28	1.21 ^d^ ± 0.11	66.49 ^aA^ ± 0.20
PS20	N.D.	68.46 ^aA^ ± 0.48	N.D.	68.16 ^aA^ ± 0.39	2.53 ^b^ ± 0.19	66.10 ^aB^ ± 0.30
PF10	N.D.	64.81 ^dA^ ± 0.26	N.D.	64.29 ^dB^ ± 0.22	0.58 ^f^ ± 0.04	64.09 ^dB^ ± 0.12
PF20	N.D.	66.49 ^cA^ ± 0.39	N.D.	66.20 ^bA^ ± 0.17	0.89 ^e^ ± 0.05	65.86 ^bB^ ± 0.15
PG10	N.D.	67.20 ^bcA^ ± 0.29	0.56 ^bB^ ± 0.05	64.16 ^dC^ ± 0.24	1.84 ^cA^ ± 0.09	65.02 ^cB^ ± 0.21
PG20	N.D.	67.64 ^abA^ ± 0.44	0.69 ^aB^ ± 0.06	65.93 ^bB^ ± 0.31	3.18 ^aA^ ± 0.11	62.98 ^eC^ ± 0.24

Explanation: CON-control sample (100% semolina durum pasta); PS—pasta with potato starch; PF—pasta with potato flakes; PG—pasta with potato grits. Data are presented as mean (*n* = 3) ± standard deviation. Lowercase letters (a–f) indicate significant differences among samples within the same column, while uppercase letters (A–C) indicate significant differences among pasta after cooking, cooling and freezing within the same sample and parameter. Different letters indicate significant differences (Tukey’s test; resistant starch, detected only after cooling and freezing, was compared using Student’s *t*-test; *p* ≤ 0.05). N.D.—not detected.

## Data Availability

The original contributions presented in this study are included in the article. Further inquiries can be directed to the corresponding author.
